# Optimising anti-seizure medication timing using a dynamic network model of seizure rhythms

**DOI:** 10.3389/fnetp.2025.1728848

**Published:** 2026-01-28

**Authors:** Jake Ahern, Udaya Seneviratne, Wendyl D’Souza, Mark J. Cook, John R. Terry

**Affiliations:** 1 Centre for Systems Modelling and Quantitative Biomedicine, University of Birmingham, Birmingham, United Kingdom; 2 Department of Neurosciences, Monash Health, Clayton, VIC, Australia; 3 Department of Neuroscience, St. Vincent’s Hospital, University of Melbourne, Melbourne, VIC, Australia; 4 Department of Medicine, St. Vincent’s Hospital, University of Melbourne, Melbourne, VIC, Australia; 5 Cornwall Intellectual Disability Equitable Research, University of Plymouth, Health and Wellbeing Innovation Centre, Truro, United Kingdom; 6 Neuronostics Ltd., Engine Shed, Station Approach, Bristol, United Kingdom

**Keywords:** anti-seizure medication, brain excitability, chronotherapy, circadian rhythms, computational modelling, epilepsy, network physiology, seizure dynamics

## Abstract

Epileptic seizures and interictal discharges exhibit robust circadian and multidien rhythms, yet the interaction between these biological cycles and anti-seizure medication (ASM) pharmacology remains poorly understood. Here, we present a dynamical network model that integrates rhythmic fluctuations in cortical excitability with pharmacokinetic properties of common ASMs to explore how treatment timing influences efficacy. The framework embeds a slow, rhythm-generating process directly within the governing equations, allowing seizure-like dynamics to emerge endogenously. We simulated ASMs with a range of distinct half-lives under single-daily and twice-daily dosing schedules. For the short half-life ASM, efficacy depended strongly on the phase of administration, with doses delivered approximately 6 h before the peak in seizure likelihood achieving up to 20% greater reduction in epileptiform discharges than suboptimal phases. In contrast, phase dependence was minimal for slower half-life drugs due to their slower elimination and flatter concentration profiles. These findings suggest that short half-life ASMs could benefit most from chronotherapeutic timing. Our framework unifies seizure dynamics, biological rhythms, and ASM pharmacology within a single model, offering a mechanistic tool to explore patient-specific optimization of treatment timing. This work establishes a foundation for translating chronotherapy into epilepsy care and provides a conceptual bridge between computational neuroscience and clinical pharmacology.

## 1 Introduction

Approximately 65 million people live with epilepsy worldwide (World Health Organisation, 2019). For many, the apparent unpredictability of seizures represents the most debilitating aspect of their condition (Schelter et al., 2008). Despite decades of research, one-third of patients remain resistant to anti-seizure medications (ASMs), and no recent drug has significantly improved this statistic (Kwan and Brodie, 2000; Chen et al., 2018). With pharmacological treatment remaining the most globally accessible therapeutic option, optimising existing ASMs is an important clinical priority.

Current ASM dosing strategies assume constant seizure risk throughout the day, which drives clinicians towards achieving relatively constant steady-state drug concentrations, typically through equally divided doses administered at regular intervals (Stanley et al., 2014). However, this paradigm increasingly contradicts emerging evidence of robust temporal organisation in seizure occurrence.

### 1.1 Seizure rhythms: from ultradian to multidien cycles

Seizures and interictal epileptiform discharges exhibit robust temporal organisation across multiple timescales, spanning from ultradian and circadian oscillations to multidien cycles of 5–30 days (Baud et al., 2018; Karoly et al., 2017; Durazzo et al., 2008; Ramgopal et al., 2014; Karoly et al., 2021b). These rhythms are not statistical anomalies but reflect fundamental properties of seizure-generating networks.

At the circadian level, many individuals demonstrate characteristic seizure “chronotypes” with seizures clustering at specific times of day that remain remarkably stable within individuals even over years (Pavlova et al., 2004; Hofstra and de Weerd, 2009). These patterns also vary systematically by epilepsy syndrome: myoclonic and myoclonic tonic-clonic seizures in juvenile myoclonic epilepsy typically occur upon awakening, whilst temporal lobe seizures often cluster in the late afternoon and evening (Zarowski et al., 2011; Seneviratne et al., 2016). Beyond daily rhythms, multidien cycles of interictal epileptiform discharges are increasingly recognised as fundamental organisers of seizure occurrence (Baud et al., 2019; Karoly et al., 2018; Reynolds et al., 2025). Analysis of long-term intracranial recordings reveals that seizures occur preferentially during the rising phase of these slower rhythms, providing a novel window into periods of heightened seizure susceptibility (Baud et al., 2018). These findings transform our understanding of seizure unpredictability–whilst individual seizures remain difficult to predict, the underlying risk appears to vary systematically and measurably over time.

### 1.2 Biological mechanisms of seizure rhythms

The mechanisms driving these temporal patterns involve multiple interacting systems. Circadian rhythms, governed by molecular clock networks in virtually every cell, create coordinated oscillations in gene expression, hormone release, and neuronal excitability (Takahashi, 2017; Bernard, 2021). Sleep-wake transitions impose additional temporal structure, with NREM sleep promoting synchrony that facilitates seizure spread whilst REM sleep provides relative protection (Dinner, 2002; Grigg-Damberger and Foldvary-Schaefer, 2015). Ultradian rhythms may reflect hormonal fluctuations, including cortisol cycles that modulate seizure threshold (van Campen et al., 2016; Marinelli et al., 2023). Slower multidien rhythms remain more mysterious, but may involve testosterone (Celec et al., 2002) (possible 20–30-day rhythms) and female reproductive cycle (25–35-day rhythm) influences (Leguia et al., 2021).

Cortical excitability is often invoked as a unifying construct to capture how diverse biological rhythms shape seizure likelihood (Badawy et al., 2012). In our framework, excitability is treated as a dynamic latent variable representing seizure propensity, rather than a directly measurable property. Many of the temporal processes outlined above can be incorporated into fluctuations of this variable, reflecting how changes in brain state modulate seizure risk. We note that excitability is not a universally defined quantity - its meaning varies across experimental methods - but recent approaches, such as the spectral slope and offset of the EEG aperiodic component, provide promising proxies for capturing these dynamics (Donoghue et al., 2020; Gao et al., 2017).

### 1.3 The possibility of chronotherapy

Despite growing recognition of seizure cycles, therapeutic exploitation of this knowledge remains limited. However, studies demonstrate that aligning ASM dosing with individual seizure patterns can significantly improve seizure control and reduce toxicity without increasing total dose. For example, patients with nocturnal seizures have shown better outcomes when the majority of their daily dose is shifted to evening hours (Yegnanarayan et al., 2006; Guilhoto et al., 2011; Thome-Souza et al., 2016). In a review, Stanley and colleagues discuss the potential for chronotherapy, highlighting studies dating back to the 1970s and propose the existence of an epilepsy “chronotype” around which treatment paradigms should be based (Stanley et al., 2014).

Practical challenges have limited chronotherapy adoption. Patient heterogeneity complicates dose scheduling, whilst complex polytherapy regimens create conflicting optimal timing requirements for different drugs.

### 1.4 Mathematical modelling as a bridge

Mathematical models offer a crucial bridge between seizure mechanisms and ASM optimisation. Traditional seizure models have focused on transitions between seizure and non-seizure states (Breakspear et al., 2006; Marten et al., 2009; Kalitzin et al., 2010; Goodfellow et al., 2011; Benjamin et al., 2012; Baier et al., 2012; Schmidt et al., 2014; Jirsa et al., 2014; Cook et al., 2022), rather than the slow modulations of seizure likelihood that occur over hours to weeks. Recent advances have incorporated multiple timescales, using a slowly varying excitability variable to represent changing seizure likelihood (Marinelli et al., 2023; Harrington et al., 2024). Other recent work has investigated how variables relevant to biological rhythms, such as melatonin, influence epileptiform activity, or have used patient-specific seizure cycles to improve seizure forecasting models (Xiong et al., 2023).

### 1.5 Study objectives

This paper extends existing models to investigate how rhythmic brain excitability interacts with ASM pharmacokinetics. By incorporating realistic drug absorption and elimination models into dynamic network seizure models, we explore how different dosing strategies influence seizure control across multiple timescales.

The approach we adopt might be particularly valuable for investigating scenarios that would be difficult or impossible to study experimentally or clinically. For example, the use of mathematical models can enable exploration of how phase relationships between endogenous excitability rhythms and medication dosing schedules influence long-term seizure control, or how individual differences in circadian timing might affect optimal dosing strategies. This mathematical framework provides a foundation for developing personalised chronotherapeutic approaches to epilepsy treatment.

## 2 Methods

### 2.1 Canonical mathematical models

Our modelling approach aims to achieve a pragmatic balance between mathematical simplicity and biological realism. As such, we utilise canonical dynamics systems: a subcritical Hopf model for describing seizure dynamics, and a van der Pol oscillator for capturing the nonlinear oscillations of time-varying cortical excitability. Each of these systems have a rich history of describing epileptiform activity dynamics (Benjamin et al., 2012; Terry et al., 2012; Woldman et al., 2019) and biological rhythms (Kronauer et al., 1982; Gonze et al., 2002; Kunz and Achermann, 2003; Creaser et al., 2021). This canonical approach enables systematic exploration of parameter space whilst preserving the nonlinear interactions between cycling excitability, network dynamics, and pharmacological perturbations that likely govern the efficacy of chronotherapeutic interventions. The resulting framework is sufficiently complex to capture biologically relevant phenomena, yet simple enough to yield mechanistic insights that can guide therapeutic strategies.

### 2.2 Rhythmic z-model structure

We start with a dynamic network model of seizure initiation. The dynamics of a brain region is described by a phenomenological model of seizure initiation that contains two states: a healthy background-like state, and a seizure-like oscillatory state. Network structure is incorporated by simulating several interacting brain regions. The equations that describe the activity of a single region are given by a modified normal form of a subcritical Hopf oscillator:

$$\frac{dz_{j}}{dt}=z_{j}\left(\lambda_{j}-1+i\omega +2|z_{j}{|}^{2}-|z_{j}{|}^{4}\right)+\alpha dW_{j}+\frac{\beta }{N}\overset{N}{\underset{k=1}{\sum }}A_{kj}\left(z_{k}-z_{j}\right),$$ (1)

where j=1,…,N represent the network nodes. The variable $z_{j}$ is a complex number, and the real part can be considered a proxy for EEG-recorded brain activity. All model variables are summarized in Table 1. The first part of Equation 1 is based upon the normal form of the subcritical Hopf oscillator. This part indicates that there are two states, one at $z_{j}=0$ which is interpreted as the background (non-discharge) state, and another at $|z_{j}{|}^{2}=1+\sqrt{\lambda_{j}}$ , in which the simulated brain activity consists of large amplitude oscillations with a period ω (the discharge state). Both seizure-like and non-seizure inter-ictal events are described by the same event discharge (ED) state. The stochastic Wiener process, $dW_{j}$ , drives the system between these two states with amplitude α . The final term describes how brain regions interact. The diffusive coupling synchronises activity by aligning nodes into the same state. The strength of the coupling is described by the scalar β . We selected a small network (N=4) with fixed coupling parameters (see Table 2) because such low-dimensional networks have previously been shown to reproduce seizure-like transitions and 24 h ED dynamics robustly while remaining computationally tractable (Marinelli et al., 2023; Terry et al., 2012).

**TABLE 1:** Model parameters and variables.

Variable	Description	Dimension
$z_{j}$	Activity of node j	2×N
$\lambda_{j}$	Excitability of node j	N
$W_{j}$	Complex Wiener process for node j	2×N
$x_{j},y_{j}$	Slow van der Pol oscillator variables	N

**TABLE 2:** Model parameters.

Parameter	Description	Value	Source
ω	Epileptiform discharge event frequency	20 Hz	Benjamin et al. (2012)
α	Noise amplitude	0.055	Benjamin et al. (2012)
β	Global coupling strength	0.35	Marinelli et al. (2023)
N	Number of nodes	4	Marinelli et al. (2023)
$A_{kj}$	Adjacency matrix	$\left(\begin{array}{cccc} 0.0 & 1.0 & 0.0 & 1.0\\ 1.0 & 0.0 & 0.0 & 1.0\\ 1.0 & 0.0 & 0.0 & 1.0\\ 0.0 & 0.0 & 1.0 & 0.0 \end{array}\right)$	Marinelli et al. (2023)
τ	Fast excitability timescale	3 s	Marinelli et al. (2023)
$\lambda_{j0}$	Baseline node excitability	0.631	This study (Section 3.1)
ρ	Rhythmic (slow) forcing amplitude	0.0014	This study (Section 3.1)
γ	Feedback from fast → slow excitability	0.0001	This study (supplementary material)
μ	Nonlinearity of slow (van der Pol) oscillator	0.01	Model choice
k	Slow oscillator timescale	$\frac{24\text{hr}}{2\pi }$ s	Model choice
$\omega_{s}$	Slow oscillator period (in units of k )	1	Model choice

The variable $\lambda_{j}$ is the equivalent of brain excitability. Large values of $\lambda_{j}$ support the oscillatory ED state, whereas low values of $\lambda_{j}$ are supportive of the steady-state background state. Excitability is modelled dynamically by

$$\tau \frac{d\lambda_{j}}{dt}=\lambda_{j0}-|z_{j}{|}^{2}-\lambda_{j}+\tau \cdot \rho x_{j}+\tau \cdot \lambda_{\mathrm{ASM}}\left(t\right),$$ (2)

$$\frac{dx_{j}}{dt}=y_{j}+\gamma \lambda_{j},$$ (3)

$$\frac{dy_{j}}{dt}=\mu \left(1-x_{j}^{2}\right)y_{j}-{\left(\frac{\omega_{s}}{k}\right)}^{2}x_{j}.$$ (4)

The first three terms of Equation 2 indicate that the excitability, $\lambda_{j}$ , tends towards the baseline $\lambda_{j0}$ and (ictal) activity induces a rapid drop in brain excitability. These dynamics ensure that seizures terminate. The timescale for fast-excitability dynamics is τ .

Slow modulation of excitability dynamics is included with the van der Pol-type system described by $x_{i}$ and $y_{i}$ in Equations 3, 4. This slow-subsystem is a (weakly) non-linear oscillator with a period $T_{s}=\omega_{s}/k$ , which we set to 24 h to represent the circadian timescale. The parameter μ controls the nonlinearity of the system; in our framework we set μ=0.01 , ensuring that the slow modulation remains close to harmonic and provides a smooth, sinusoidal-like forcing of the fast excitability dynamics. The rhythmicity parameter, ρ , parameterises the interaction of the slow system onto the fast system. Larger values of ρ result in a stronger rhythmic drive. The excitability can also interact with the slow system via the feedback parameter γ . The absence of feedback (γ=0) indicates that the excitability dynamics, including the effect of ASMs, does not impact the slow rhythm. This may be the case if, for example, the slow oscillation was driven by hormonal rhythms. In the presence of feedback (γ>0) , excitability dynamics can alter the slow rhythm. In this case, excitability could be modulated by a cell-intrinsic rhythm in cellular excitability, for example, (see Supplementary Material). Finally, ASM in this framework is modelled as a perturbation to the excitability (see Section 3.2).

This model incorporates noise-driven seizure initiation and termination, brain network structure, slow modulation of brain excitability, and the effect of ASM into a unified framework. Similar versions of this model has been used to understand the brain’s response to ASMs (Woldman et al., 2020; Harrington et al., 2024), the difference between types of epilepsy (Terry et al., 2012), the robustness of surgical treatment (Junges et al., 2020) and potential drivers of circadian variability in ED (Marinelli et al., 2023). With the framework presented above we can investigate how features of dynamic brain networks, biological rhythms and their perturbations result in rhythmic patterns of epileptic activity.

### 2.3 Numerical methods

All simulations were implemented in Python (version 3.12.4) using the scientific computing libraries NumPy (v1.26.4), SciPy (v1.13.1), pandas (v2.2.2), and matplotlib (v3.8.4). The system of differential equations was solved using the Euler-Maruyama method with a fixed step size of dt=0.001 . Results are reported as averages of 10 independent runs to account for stochastic variability. Simulations were performed on the BlueBEAR High Performance Computing cluster at the University of Birmingham. A typical 30-day simulation required approximately 100 min of wall-clock time.

## 3 Results

### 3.1 Emergence of ED cycles

**FIGURE 1:**
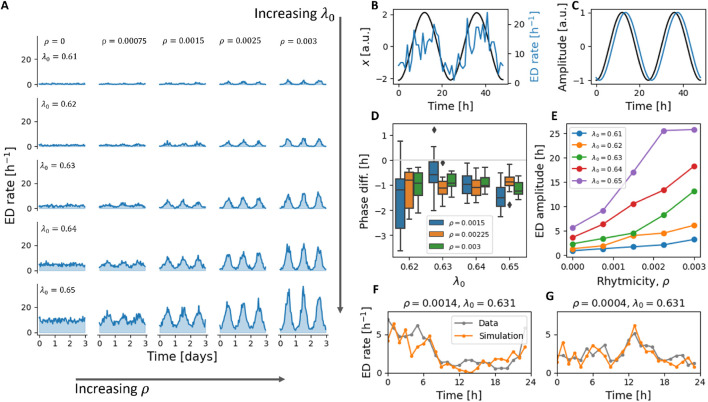
Spontaneous ED activity. **(A)** Variation of the rhythmicity strength, ρ , and the baseline excitability, $\lambda_{0}$ , results in a diverse array of activity patterns. Simulation parameters are the same as Table 2. **(B)** Representative plot showing the hourly ED rate (blue) and the slow forcing variable, x (averaged over all nodes). **(C)** Two cosinor functions fitted to the data in **(B)** are plotted again with normalised amplitude and mean to illustrate the small phase difference between the rhythms. **(D)** The phase difference between x and the ED rhythm across $\lambda_{0}$ and ρ values. **(E)** The amplitude of the ED signal plotted against ρ , for different $\lambda_{0}$ values. **(F,G)** The hourly ED rate obtained from EEG data (Seneviratne et al., 2016), split into two ED-chronotypes (as in Marinelli et al. (2023)) is plotted in grey. Best fit simulation results are plotted in orange.

We begin by simulating spontaneous ED activity in a four-node network with varying values of rhythmicity strength (ρ) and baseline excitability $\left(\lambda_{0}\right)$ . No medication is administered and $\lambda_{\mathrm{ASM}}=0$ . When ρ>0 , the model exhibited emergent daily oscillations in hourly ED rate (Figure 1A). As expected, the amplitude of these oscillations increased with ρ , indicating stronger rhythmic modulation (Figure 1E). In contrast, decreasing $\lambda_{0}$ resulted in a lower mean ED rate and a dampening of the oscillation amplitude, reflecting reduced overall excitability (Figure 1E).

To investigate the temporal relationship between brain excitability and the ED cycle, we fitted cosinor functions to both the slow forcing variable x and the hourly ED histograms over the final 2 days of 15-day simulations. The phase difference between these two rhythms was then computed (illustrated in Figures 1B,C). Across conditions that produced sufficiently rhythmic ED time series, the mean phase difference was −1.04±0.28 hours, indicating that changes in excitability preceded changes in ED activity by approximately 1 h. Variation in $\lambda_{0}$ and ρ did not significantly affect the phase difference, nor did their interaction. There was a trend toward an effect of $\lambda_{0}$ (p = 0.066, ANOVA), but stochastic variability across simulations accounted for most of the variance (Figure 1D).

To assess the biological plausibility of our model-generated ED cycles, we compared simulation output to EEG recordings from the dataset in Seneviratne et al. (2016) ( n≈100 , 24-h duration). This dataset was previously used to define two empirical ED chronotypes: a group with peak EDs during sleep (SLEEP group); and a group with peak EDs during daytime hours (presumed to align with cortisol rhythms–CORT group). We conducted a grid search over ρ and $\lambda_{0}$ and identified the best-fit parameter pairs for each group via least-squares optimization. The resulting simulated histograms matched the empirical ED distributions (Figures 1D,E), with reasonable agreement for both the SLEEP $\left(R^{2}=0.57\right)$ and CORT $\left(R^{2}=0.27\right)$ groups. The primary difference between groups was the estimated rhythmicity strength: $\rho_{\mathrm{SLEEP}}=0.0014$ and $\rho_{\mathrm{CORT}}=0.0004$ . These results suggest that group-level ED timing patterns can be reproduced by our framework and explained by differences in rhythmic modulation of excitability.

### 3.2 Modelling ASM effects on excitability

**FIGURE 2:**
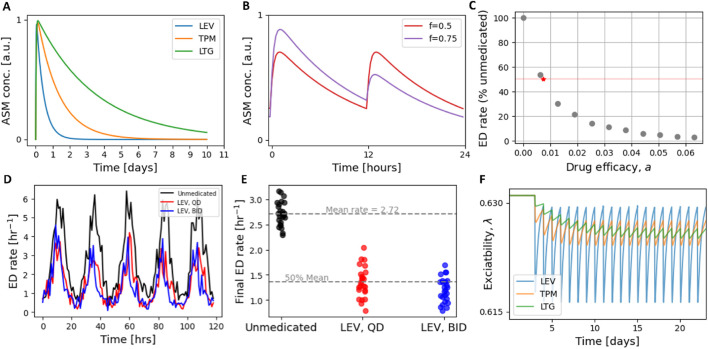
Modelling ASM effects. **(A)** The effect-site concentration profile of three different ASMs with a dose administered at t=0 (see Equation 5). **(B)** Concentration profile of LEV under two different BID schedules: an even split of the daily dose (red), and an 75/25 split (purple). **(C)** An example sweep of 10 efficacy parameters for QD LEV treatment. The final ED rate is shown as a percentage of the unmedicated ED rate. A value of a=0.0073 aligns with a 50% reduction (red star). **(D)** The hourly ED rate for a network with daily ED rhythms under three different treatment schemes: unmedicated (black); medicated with a single daily dose of LEV (red); and medicated with the same total dose but split equally over two dose times (blue). Doses were administered across a range of ED cycle phases and averaged. **(E)** The mean ED rate from simulations in **(D)**. Treatment with LEV (a=0.0073) results in a 50% reduction, on average, under QD schedules and slightly less for BID dosing (45.6%). **(F)** An example QD schedule for LEV (blue; $a_{\mathrm{LEV}}=0.0073$ ), TPM (orange; $a_{\mathrm{TPM}}=0.002$ ) and LTG (green; $a_{\mathrm{LTG}}=0.00078$ ).

The effect of ASMs in the dynamical network model is represented as a transient reduction in brain excitability. The waveform of the excitability perturbation caused by a single dose is based on a simple pharmacokinetic model of effect-site concentration. We assume the ASM-induced reduction in excitability is proportional to the drug concentration. The change in concentration following a single dose is written as

$$\text{ASM}\left(\eta \right)=C\;\left(e^{-k_{e}\eta }-e^{-k_{a}\eta }\right),$$ (5)

where η is the time elapsed since administration, $k_{a}$ and $k_{e}$ are effective absorption and elimination rates, respectively, and C is a normalizing constant such that a single dose reaches a maximum amplitude of one (see Figure 2A). In words, drug concentration initially rises with rate $k_{a}$ (absorption) and then returns toward baseline with rate $k_{e}$ (elimination). The parameters $k_{a},k_{e}$ for each ASM considered are given in Table 3.

**TABLE 3:** ASM parameters.

Parameter	Description	Value	Source
$a_{\mathrm{LEV}}$	LEV efficacy parameter	0.0073	This study (Section 3.2)
$k_{a,LEV}$	LEV absorption rate	2.618/3600 ${\text{s}}^{-1}$	Besné et al. (2025), Iapadre et al. (2018)
$k_{e,LEV}$	LEV elimination rate	0.099/3600 ${\text{s}}^{-1}$	Besné et al. (2025), Iapadre et al. (2018)
$a_{\mathrm{TPM}}$	TPM efficacy parameter	0.002	This study (Section 3.2)
$k_{a,TPM}$	TPM absorption rate	1.215/3600 ${\text{s}}^{-1}$	Besné et al. (2025), Iapadre et al. (2018)
$k_{e,TPM}$	TPM elimination rate	0.033/3600 ${\text{s}}^{-1}$	Besné et al. (2025), Iapadre et al. (2018)
$a_{\mathrm{LTG}}$	LTG efficacy parameter	0.00078	This study (Section 3.2)
$k_{a,LTG}$	LTG absorption rate	1.572/3600 ${\text{s}}^{-1}$	Besné et al. (2025)
$k_{e,LTG}$	LTG elimination rate	0.012/3600 ${\text{s}}^{-1}$	Besné et al. (2025)

For multiple doses, the net ASM perturbation is the sum of the contributions from each administered pulse. In schedules with two daily doses (BID), if the first (primary) dose is given at time $t_{i}$ and the secondary dose at $t_{i}+\tau$ (here and throughout the rest of this study, τ=12 hours), and we define f∈[0,1] as the fraction of the total daily dose delivered in the primary dose (e.g., f=0.5 for a 50/50 split, Figure 2B), the total drug-induced perturbation at time t is

$$\lambda_{\text{ASM}}\left(t\right)=-a\;\underset{i}{\sum }\left[f\cdot ASM\left(t-t_{i}\right)+\left(1-f\right)\cdot ASM\left(t-t_{i}-\tau \right)\right],$$ (6)

where ASM(⋅) denotes the effect-site concentration profile. For single daily dosing (QD) schedules, f=1 . The time taken to achieve a steady-state average concentration depends upon the timescale of the ASM - compounds with a longer half life take longer to reach steady state (Figure 2F) In all simulations, the ASM concentration achieved an average steady state before measurements were taken.

#### 3.2.1 Calibration of a

We treated a as a drug-specific efficacy parameter mapping concentration to excitability reduction. For comparability across drugs and schedules, a was calibrated so that under the single daily dose (QD) control condition each drug produced an approximately 50% reduction in mean ED rate relative to the unmedicated baseline. The QD control was defined as the mean outcome across several evenly spaced dose phases, representing typical clinical administration independent of seizure phase.

We selected three commonly used ASMs with distinct pharmacokinetic properties: levetiracetam (LEV, half-life ∼ 7 h), topiramate (TPM, half-life ∼ 21 h), and lamotrigine (LTG, half-life ∼ 56 h). Throughout this study, we modelled monotherapy only; that is, each simulation considered the effect of a single ASM in isolation. In our framework, all drugs were modelled phenomenologically as transient reductions in excitability, differing only in the timescale of their pharmacokinetics. We note that LTG half-life values reported in the literature vary substantially (typically 25–40 h depending on formulation and population) (Iapadre et al., 2018). Here, LTG is used solely as a stand-in for a slow-elimination ASM, and our choice of a ∼ 56 h half-life follows a pharmacokinetic dataset used for parametrisation (Besné et al., 2025). Because our model does not incorporate drug-specific mechanisms, only relative elimination timescales matter for the presented results.

For each drug, we performed a sweep over a (including a=0 , i.e., unmedicated) in the QD control schedule and simulated until λ(t) reached stable periodic behavior for at least 3 days. The mean hourly ED rate over the final 3 days was recorded for each a , and we selected the value that produced an approximately 50% reduction in mean ED rate. These calibrated values are reported in Table 3. Briefly, longer half-life drugs required smaller a values because their slower elimination leads to greater accumulation across repeated doses, yielding a higher steady-state concentration. As a result, while all drugs were calibrated to the same 50% efficacy under control dosing, their dynamical effects differ: shorter half-life compounds produce relatively large fluctuations in excitability, whereas longer half-life compounds generate smoother, lower-amplitude perturbations once steady state is reached (Figure 2F).

We retained the QD-calibrated a values as drug-specific efficacy parameters in subsequent experiments. Applying the same a to BID control dosing produced approximately 50% reductions (53.3%, 49.6%, and 50.8% for LEV, TPM, and LTG, respectively).

### 3.3 ASM dose timing modulates seizure likelihood

**FIGURE 3:**
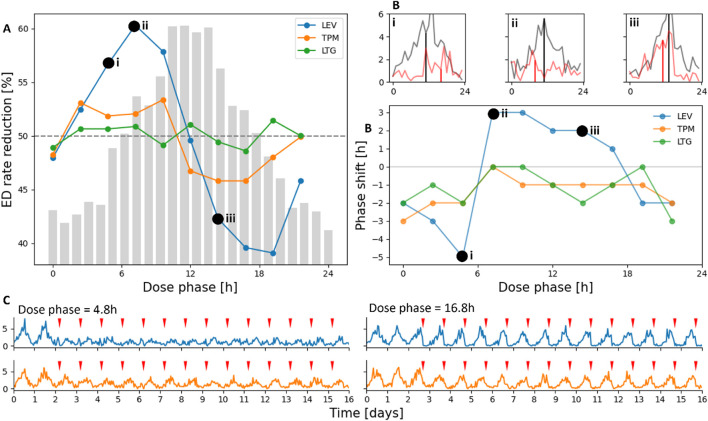
ASM dose timing modulates seizure likelihood. **(A)** Final ED rate (mean over last 7 days) after treatment with LEV, TPM, and LTG at different dose phases. The grey histogram illustrates a representative ED cycle. **(B)** Phase response curve showing the phase shift of the ED rhythm between the first (grey) and final (red) day of dosing. Insets i-iii show ED histograms on the first and last day for three different dose phases; vertical lines mark cosinor-derived peaks. **(C)** Example hourly ED rates for LEV (blue) and TPM (orange).

We now examine how ASM dose timing interacts with ED rhythms. In these simulations, the model generated 24-h excitability fluctuations that produced ED cycles, with a peak at 12 h. Model parameters are in Table 2, with $\lambda_{0}=0.631$ and ρ=0.0014 . After 2 days of unmedicated activity, a single daily ASM dose (QD schedule, Figure 3A) was administered for 28 days. The three ASMs (LEV, TPM and LTG) were tested, with ten evenly spaced dosing phases across the circadian cycle.

Treatment efficacy was measured as the percentage reduction in mean hourly ED rate over the final 7 days compared to the unmedicated condition. For LEV, efficacy depended strongly on dose phase: optimal dosing occurred near 6 h (rising phase of the ED rhythm), reducing the ED rate by 60% – a 10% improvement over the control condition. In contrast, dosing during the falling phase reduced ED rate by only 39% (Figure 3A), leading to a 21% difference in efficacy solely from timing. Notably, these gains required no change in dose amount, only timing.

By contrast, TPM and LTG exhibited minimal phase dependence, with efficacy ranging from 46% to 53% and 49%–52%, respectively (Figure 3A). Their slower pharmacokinetics produce flatter concentration profiles, so dose timing exerts little influence on the temporal pattern of excitability perturbation (Figure 2F). These findings suggest that circadian ED patterns may be more effectively targeted using short half-life ASMs administered ∼ 6 h before peak ED likelihood, whereas longer half-life compounds provide more uniform but less phase-sensitive control.

Finally, LEV dosing also induced phase shifts in the ED rhythm (Figure 3B). Doses near the cycle trough delayed the rhythm, whereas doses near the peak advanced it by up to 6 h. Such shifts were much less pronounced for TPM and LTG, which generally delayed the ED cycle by an hour. This is consistent with their smaller concentration fluctuations.

### 3.4 BID dosing schedules

We next extended the analysis to twice-daily dosing (BID), which more closely reflects current clinical practice. The simulation setup was identical to the QD analysis, except two doses per day were delivered. We explored how the phase of the primary dose influenced efficacy by simulating ten equally spaced phases across the circadian ED cycle. We also varied the fraction of the daily dose delivered in the primary dose (f) , from 0.5 to 0.9, while keeping the total daily dose constant (see Equation 6; Supplementary Figure S4).

**FIGURE 4:**
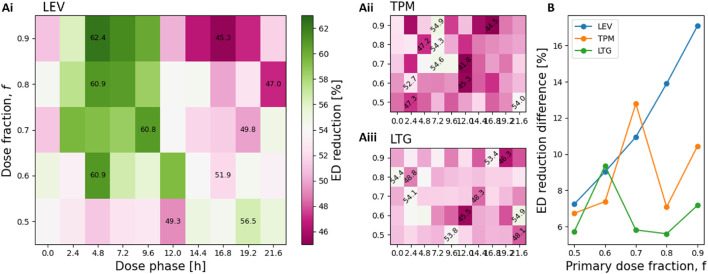
ASM dose timing and dose fraction modulate treatment efficacy. **(A)** Heatmaps of ED reduction for BID schedules with different primary dose fractions (rows) and dose phases (columns). The maximum and minimum values for each row are printed at their corresponding coordinates. **(B)** Difference between maximum and minimum ED reduction for each dose fraction, shown separately for LEV, TPM, and LTG.

These findings extend naturally from the QD results. For LEV, the largest reduction was observed when most of the daily dose (f=0.9) was administered ∼ 6 h before peak ED activity, reducing EDs by 62.4% (Figure 4Ai). Conversely, dosing ∼ 6 h after the ED peak gave the poorest efficacy (45.3%). As f decreased, excitability fluctuations became smaller and steadier, and the dependence of efficacy on dose phase diminished. Figure 4B illustrates this by plotting the difference between the most and least effective schedules for each f : for LEV, this range grows with increasing f , highlighting the importance of timing for short half-life drugs.

For the slower-acting drugs TPM and LTG, efficacy showed little relation to either dose phase or fraction (Figures 4Aii,iii), consistent with their flatter concentration profiles.

## 4 Discussion

### 4.1 Key findings and mechanistic insights

A key finding is that endogenous biological rhythms fundamentally alter the effectiveness of anti-seizure medications through phase-dependent interactions in our theoretical framework. Short half-life ASMs, such as levetiracetam, provide substantially greater benefit when dosing is aligned with simulated seizure cycles, with optimal timing occurring approximately 6 hours before peak epileptiform discharge activity. Crucially, this improvement arises solely from adjusting dose timing - no increase in total dose is required.

This work advances our mechanistic understanding by demonstrating how rhythms emerge naturally from coupled excitability dynamics. Unlike previous models that imposed seizure cycles externally (Marinelli et al., 2023), our framework embeds the rhythm-generating system directly within the governing equations, providing a self-consistent description of how endogenous biological processes might shape seizure likelihood. The model successfully reproduces empirically observed chronotypes and predicts the phase relationships between excitability fluctuations and epileptiform activity.

The framework also integrates ASM pharmacokinetics directly into seizure dynamics, revealing why timing matters for some drugs but not others in our simulations. Short half-life compounds produce sharp fluctuations in brain excitability that can be strategically aligned with natural vulnerability windows, whilst longer half-life drugs generate steady but less optimisable coverage.

### 4.2 Implications for network physiology

Network Physiology is an emerging field that examines how physiological systems dynamically interact across multiple spatiotemporal scales to coordinate function (Ivanov, 2021; Bartsch et al., 2015). By integrating brain dynamics with biological rhythms and pharmacokinetic processes, our framework considers the multi-system interactions that define seizure emergence. Rather than being considered in isolation, here the brain operates as a key node within a broader physiological network. From a network physiology perspective, the rhythmic modulation of seizure susceptibility could be considered “physiological coupling”, that is dynamic interactions between systems that enable coordinated function. In our case, cortical excitability serves as an interface between circadian timing networks, seizure-generating circuits, and pharmacological perturbations.

Further, chronotherapy may be considered a network-based therapeutic approach that enhances physiological resilience by working with, rather than against, the natural temporal organisation of the network. The differential timing effects we observed for short versus long half-life medications reflect how therapeutic interventions interact with network dynamics: interventions aligned with natural network rhythms can leverage inherent physiological coordination mechanisms for enhanced efficacy. This principle extends beyond epilepsy to suggest that effective therapeutics should account for the temporal organisation and coupling characteristics of physiological networks. Future developments integrating wearable monitoring with network-based models could enable real-time assessment of physiological network states and dynamic therapeutic optimisation. Such an approach could represent a paradigm shift towards truly personalised, network-informed medicine. The implications of which we discuss in the next section.

### 4.3 Possible clinical implications

These theoretical findings suggest possible clinical relevance that would require careful validation. For patients taking short half-life ASMs like levetiracetam, redistributing doses to align with seizure patterns could potentially improve outcomes without increasing medication burden or side effects. Our model predictions are consistent with limited existing clinical reports showing that differential dosing strategies can improve seizure control in selected patient populations.

Specifically, Guilhoto et al. (2011) demonstrated that higher evening dosing in 17 paediatric patients with nocturnal seizures led to seizure freedom in 64.7 % of patients, with 88.2 % experiencing ≥50% seizure reduction after a mean follow-up of 5.3 months. Similarly, Thome-Souza et al. (2016) showed that higher-evening differential dosing of clobazam as add-on therapy resulted in a median seizure reduction of 75 % compared to 50 % in controls (p<0.005) , with patients tolerating higher total daily doses without increased adverse events. It should be noted though that these studies were small, retrospective and focused on specific patient populations, highlighting the need for more robust clinical validation.

Recent reviews have emphasised the potential of chronotherapy in epilepsy management. Khan et al. (2018) noted that circadian rhythms can shape temporal patterns of epileptic seizures and suggested that timing of antiepileptic drug administration could be optimised based on individual seizure patterns. Næsgaard et al. (2023) reviewed the principles of differential dosing, emphasising that tailoring drug concentration to seizure timing patterns represents a promising but underexplored therapeutic approach. Niu et al. (2025) highlighted chronotherapy as a promising approach for optimising epilepsy management by aligning treatment schedules with biological rhythms, though they noted clinical implementation remains challenging.

A possible framework to address the topic would be a three-phase approach. Phase 1 would be a prospective observational study to explore more robustly the relationship between natural variation in timing of dosing and seizure outcomes in people already taking short half-life ASMs. This would require long-term seizure diary collection, augmented by a detailed log of dose timing and adherence. There are important caveats on the reliability of seizure diaries (Reynolds et al., 2025), however, such a study would be an important first step towards identifying people with epilepsy with clear rhythmic seizure patterns.

Following observational validation, a small randomised controlled trial could test timing of dosing in carefully selected patients. Inclusion criteria for such a study might include people on levetiracetam monotherapy, with excellent adherence, and whom have clear rhythmic seizure patterns documented over 3 months or longer. The primary endpoint would be change in seizure frequency over a 6-month intervention period, with secondary endpoints including seizure severity, quality of life measures, medication side effects, and sleep quality. A crossover design would minimise confounding and permit within-patient comparisons, whilst rigorous safety monitoring with a clear stop criteria would be needed should seizures worsen.

Finally, should the proof-of-concept phase show promise, a much larger multicentre trial could evaluate broader patient populations, multiple short half-life ASMs, longer study window (12 months plus) and health-economic outcomes. In all studies, it is important to carefully consider the ethical implications of interventions that incur the possibility of worsening seizure control.

The framework could ultimately support personalised treatment by predicting optimal dosing windows for candidate ASMs, but this would require extensive validation against real-world outcomes and careful consideration of individual patient factors including chronotype, seizure patterns, and pharmacokinetic variability.

### 4.4 Model limitations

Several simplifications constrain the biological realism of our framework and limit direct clinical translation. The epileptiform discharge-generating system was deliberately kept simple to enable analytical insight (Benjamin et al., 2012), but this may limit its capacity to reproduce the detailed structure of empirically observed rhythms. More mechanistic formulations incorporating explicit excitatory-inhibitory interactions (Cook et al., 2022; Jirsa et al., 2014) could better capture network dynamics and facilitate modelling of polytherapy scenarios that reflect real clinical practice.

Further, ASM effects were modelled as transient, concentration-dependent reductions in excitability. Whilst this keeps the model tractable, it inevitably abstracts away the pharmacological diversity of real ASMs, which act via distinct mechanisms including sodium channel blockade, GABA enhancement, and calcium channel modulation (Johannessen Landmark et al., 2023; Bialer et al., 2024). The single-compartment pharmacokinetic model also ignores circadian modulation of drug absorption, distribution, metabolism, and elimination, which could significantly influence optimal timing predictions (Ramgopal et al., 2013).

Another limitation is our assumption of perfect adherence. In reality, missed doses and timing variability are common, with studies showing adherence rates of 58–86 % in epilepsy patients (Terman et al., 2021). Poor adherence poses substantial practical obstacles for chronotherapy implementation and could negate any theoretical benefits of optimised timing (Stanley et al., 2014; Ramgopal et al., 2013). Additionally, we did not model side-effect profiles, which may vary with dosing schedules and could influence the risk-benefit calculation for individual patients (Yegnanarayan et al., 2006; Ben-Cherif et al., 2012).

Finally, model predictions are based on theoretical seizure cycles that may not accurately reflect the complexity and heterogeneity of real epilepsy syndromes. Individual variations in seizure patterns, drug metabolism, and underlying pathophysiology could significantly alter the effectiveness of timing-based interventions (Ramgopal et al., 2013; Gesche and Beier, 2022). Furthermore, our framework assumes stable, predictable circadian rhythms, whereas real patients may experience rhythm disruption due to shift work, sleep disorders, or other medical conditions (Smith et al., 2025; Cheng et al., 2024).

### 4.5 Future directions

Three key developments would advance this theoretical framework toward potential clinical application. First, patient-specific calibration of the excitability-drug concentration relationship could enable personalised predictions of treatment efficacy. This mapping could potentially be grounded empirically through EEG-based measures of cortical excitability (Meisel et al., 2015), transcranial magnetic stimulation protocols (Badawy et al., 2014), or novel biomarkers derived from wearable devices (Karoly et al., 2021a), though the relationship between our theoretical excitability variable and measurable biomarkers would require extensive validation.

Second, integration with wearable physiological monitoring could provide real-time assessment of seizure risk and optimal medication timing (Liu et al., 2022). Recent advances in consumer wearables have shown promise for detecting seizure-related and ASM-related physiological changes (Ahuja et al., 2024; Halimeh et al., 2022; Halliday et al., 2025), and machine learning approaches have shown promise for predicting seizure likelihood from multimodal physiological data (Karoly et al., 2017). This closed-loop approach represents a long-term goal for personalised chronotherapy, where treatment decisions adapt continuously based on brain state, but would require extensive safety validation and regulatory approval. Additionally, patient-specific EEG data could be used to extract region-specific features, such as alpha power or other spectral markers, which could then inform node-specific parameters and enable larger, individualized network models.

Finally, extension to polytherapy scenarios would address real clinical practice, where 30–40 % of patients take multiple ASMs with potentially conflicting optimal timing requirements (Chen et al., 2018). Understanding how different drug mechanisms interact with biological rhythms could inform rational combination strategies (Manganaro et al., 2017), though this adds considerable complexity to both modelling and clinical implementation. Additionally, network pharmacology approaches may provide frameworks for understanding these complex interactions (Harrington et al., 2024).

## 5 Conclusion

We have demonstrated that even a minimal theoretical model linking biological rhythms and ASM pharmacology can reveal potential strategies for treatment timing. The core principle suggests that we might improve seizure control by working with the brain’s natural rhythms rather than against them (Stanley et al., 2014). For short half-life medications, strategic timing provides a theoretical pathway to better outcomes without increasing medication burden.

Our framework proposes a theoretical foundation for chronotherapy in epilepsy and identifies the key parameters that determine when timing-based interventions might be beneficial. Extending this approach with patient-specific modelling and rigorous real-world validation could potentially contribute to more personalised seizure management. This could offer new avenues for investigation in the treatment of millions of people whose seizures remain poorly controlled despite optimal conventional treatment.

## Data Availability

The EEG data analyzed in this study were previously collected from individuals with epilepsy. The data are not publicly available due to participant privacy considerations and institutional ethical restrictions. Requests to access these datasets should be directed to Udaya Seneviratne, Udaya.Seneviratne@monash.edu.
