# Supplementary material for: Optimising anti-seizure medication timing using a dynamic network model of seizure rhythms
Source: Front Netw Physiol. 2026 Jan 28;5:1728848. doi: 10.3389/fnetp.2025.1728848 (PMC12891084; doi:10.3389/fnetp.2025.1728848)
Supplement: Supplementary file 1 [file DataSheet1.pdf]

Supplementary Material

for *Optimizing anti-seizure medication timing using a dynamic network model of rhythmic seizures*

Jake Ahern, Udaya Seneviratne, Wendyl D'Souza, Mark J. Cook and John R. Terry

1 Exploring the effects of rhythmicity strength, ρ, and slow feedback, γ

Biological interpretation of the feedback parameter, γ

The feedback strength parameter, γ, represents coupling from the fast excitability system (seizure and ASM dynamics) back to the slow rhythm-generating process. Biologically, this captures potential bidirectional interactions between acute neural activity and the slower physiological or molecular mechanisms that modulate excitability over longer timescales.

A nonzero γ reflects the hypothesis that seizure activity and ASM effects can influence the generators of slow excitability rhythms. Several biological pathways could underlie such feedback. Seizures have been shown to alter the expression and phase of core clock genes such as *Per1*, *Per2*, and *Bmal1*, indicating direct cross-talk between neuronal activity and circadian machinery (Matos et al., 2018; Eun et al., 2011; Wallace et al., 2018; Gerstner et al., 2014). In addition, activity-dependent homeostatic mechanisms may operate on similar timescales and can gradually adjust baseline excitability. Neuroendocrine and immune responses to seizures or ASM exposure may also provide additional pathways for slow feedback.

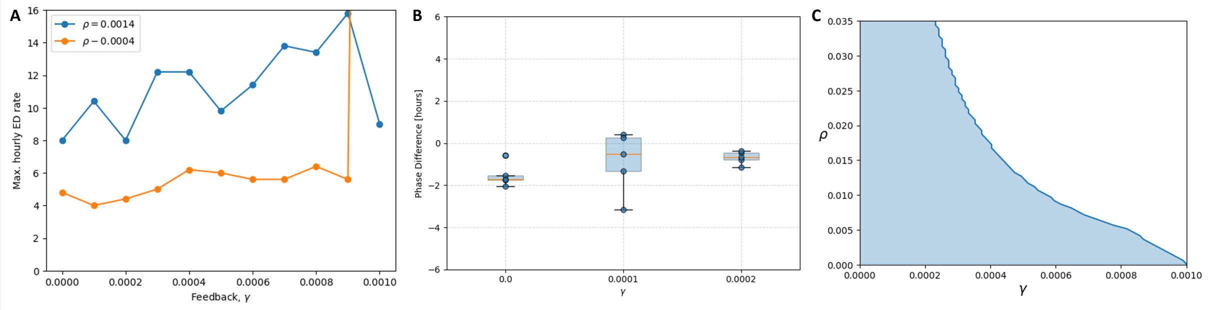

Figure 1: (A) Increasing γ increases the amplitude of the ED cycle. (B) The phase difference between average excitability and hourly ED rate for three feedback strengths (ρ adjusted to maintain invariant λ oscillations; see next figure). (C) The region of (ρ, γ) space in which stable λ oscillations exist.

However, γ need not always be nonzero: some slow modulators, such as reproductive hormone cycles, may act independently of acute seizure dynamics, implying a primarily unidirectional influence from the slow to the fast system (Alshakhouri et al., 2024; Foldvary-Schaefer et al., 2004).

One initial aim of this study was to test whether varying γ influenced spontaneous seizure cycles or ASM treatment outcomes. Simulations showed that γ had a negligible impact on both measures; therefore, these analyses were omitted from the main text. Despite its minimal effect at the circadian timescale investigated here, we retain a small but nonzero value ($\gamma = 0.0001$) to capture weak yet plausible bidirectional coupling, while keeping the model general enough to apply to systems in which feedback may become more relevant at longer timescales.

Choice of the feedback strength parameter, γ

We first examined how varying γ influenced ED cycle amplitude in the full model (Equations 1–4). For both low ($\rho = 0.0004$) and high ($\rho = 0.0014$) rhythmicity, increasing γ proportionally increased the amplitude of the slow oscillation (x) and, consequently, the ED rhythm amplitude (Supplementary Figure 1A). However, the phase relationship between excitability and ED rate, as well as overall rhythmic behaviour, remained unchanged (Supplementary Figure 1B).

We then explored the combined effects of ρ and γ in the reduced system:

$$\tau \frac{d\lambda_j}{dt} = \lambda_{j0} - \lambda_j + \tau \cdot \rho x_j, \tag{1a}$$
$$\frac{dx_j}{dt} = y_j + \gamma \lambda_j, \tag{1b}$$
$$\frac{dy_j}{dt} = \mu(1 - x_j^2) y_j - \left(\frac{\omega_s}{k}\right)^2 x_j, \tag{1c}$$

which correspond to Equations (2–4) in the main text with $z_j = 0$, ASM$(t) = 0$, and no seizure activity. Increasing ρ or γ beyond moderate values caused λ to diverge, allowing us to map the region in (ρ, γ) space where stable oscillations existed (Supplementary Figure 1C). All (ρ, γ) combinations used in the main text lie within this stable region.

For nonzero γ, increasing ρ also increased the mean of the excitability oscillation. Supplementary Figure 2A,B shows heatmaps of the excitability oscillation mean and amplitude across (ρ, γ) space. Altering γ thus affects both the amplitude and mean of λ oscillations, which can be compensated by adjusting ρ and λ_0, respectively.

To compare pharmacological simulations across different γ values, we required systems with distinct feedback strengths but identical λ oscillations. Keeping λ_0 constant, we identified (ρ, γ) pairs at the intersection of the level sets in Supplementary Figure 2A,B (see panel C). The resulting oscillations are shown in panel D. For further analyses, we used $\gamma = 0, 0.0001, 0.0002$.

When evaluating pharmacological simulations, varying γ across these values produced nearly identical results for both spontaneous activity and ASM dose–phase sweeps (Supplementary Figure 3). We therefore set $\gamma = 0.0001$ for all simulations in the main text, representing an intermediate value that captures weak feedback coupling without altering the rhythmic structure or treatment outcomes.

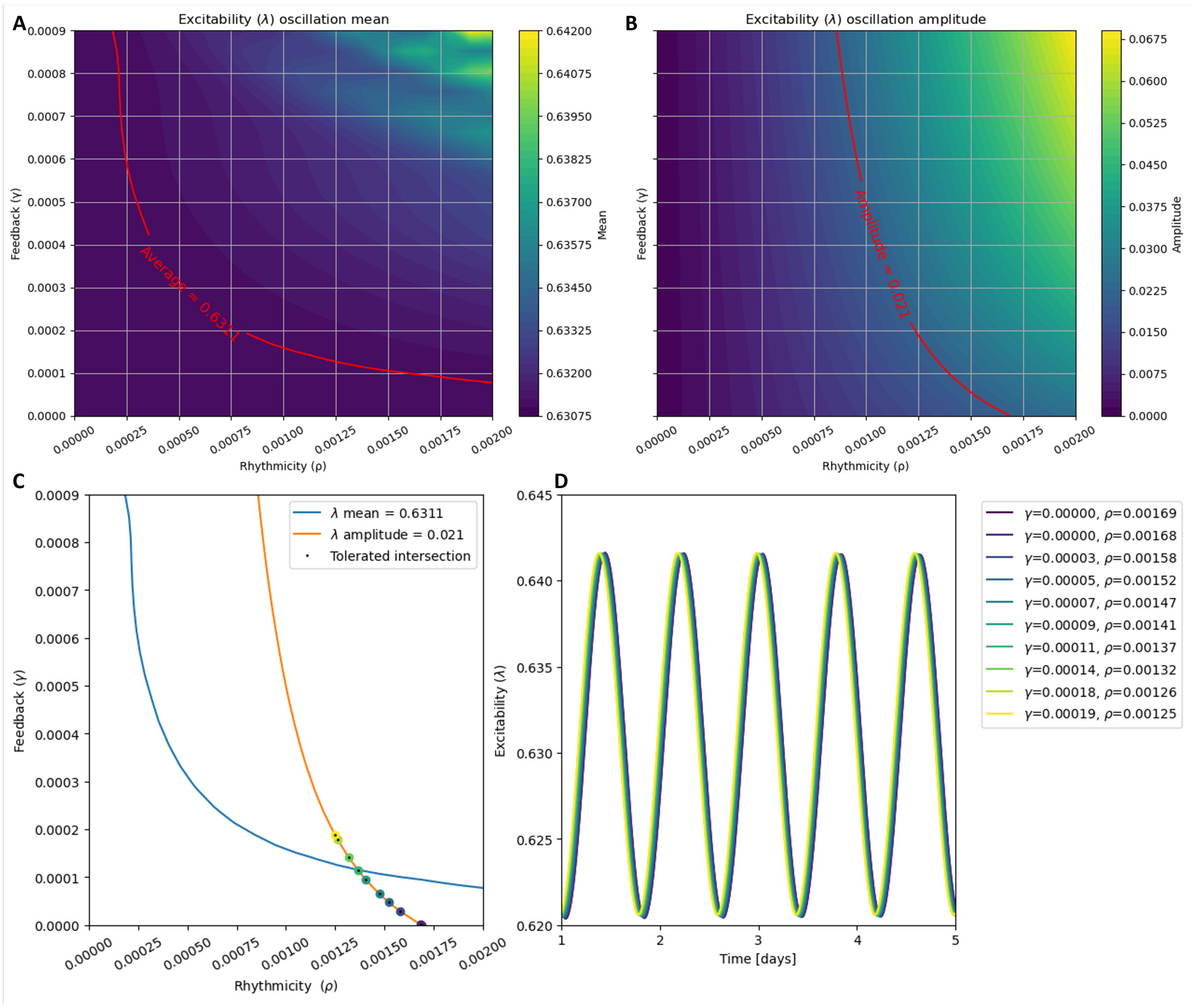

Figure 2: Heatmaps showing the excitability oscillation mean (A) and amplitude (B) across a 20×20 grid of ρ and γ values. Level sets (red) denote target values. (C) The intersection of the two level sets identifies (ρ, γ) pairs yielding consistent oscillations. (D) Simulated excitability (λ) dynamics for selected (ρ, γ) combinations.

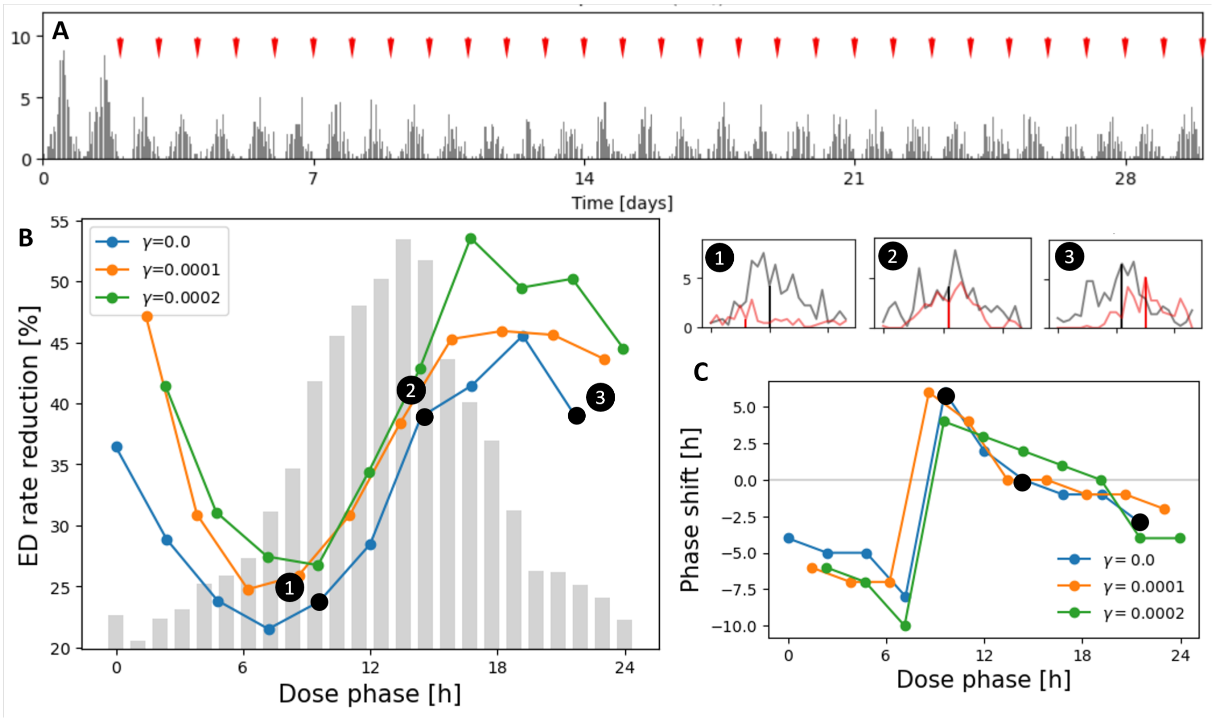

Figure 3: ASM dose timing modulates seizure likelihood. (A) Dosing protocol showing 10 regular dose phases across the ED cycle (red arrows). (B) Final ED rate (mean over the last 7 days) after LEV treatment at each dose phase. The grey histogram represents a typical ED cycle. Insets 1–3 show ED histograms on the first (grey) and last (red) day; vertical lines mark the cosinor-derived peaks. (C) Phase response curve showing the phase shift between the first and last day of the ED rhythm.

1.1 Different dosing schedules have the same area under the curve

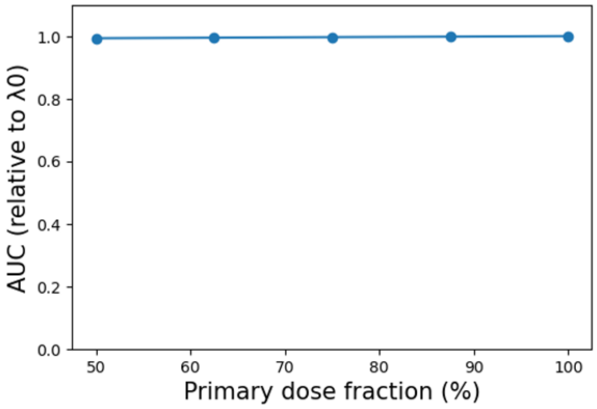

Figure 4: The area under the excitability curve (relative to λ_0) for multiple dosing schedules. The curve represents excitability dynamics with $\rho = 0$ driven by ASM concentration profiles. All BID schedules, including QD, have equivalent area under the curve and therefore deliver the same total drug exposure.

References

Alshakhouri, M., Sharpe, C., Bergin, P., and Sumner, R. L. (2024). Female sex steroids and epilepsy: Part 1. A review of reciprocal changes in reproductive systems, cycles, and seizures. *Epilepsia* 65, 556–568. doi:10.1111/epi.17842

Eun, B., Kim, H. J., Kim, S. Y., Kim, T. W., Hong, S. T., Choi, K. M., et al. (2011). Induction of Per1 expression following an experimentally induced epilepsy in the mouse hippocampus. *Neuroscience Letters* 498, 110–113. doi:10.1016/j.neulet.2011.03.039

Foldvary-Schaefer, N., Harden, C., Herzog, A., and Falcone, T. (2004). Hormones and seizures. *Cleveland Clinic Journal of Medicine* 71 Suppl 2, S11–18. doi:10.3949/ccjm.71.suppl_2.s11

Gerstner, J. R., Smith, G. G., Lenz, O., Perron, I. J., Buono, R. J., and Ferraro, T. N. (2014). BMAL1 controls the diurnal rhythm and set point for electrical seizure threshold in mice. *Frontiers in Systems Neuroscience* 8, 121. doi:10.3389/fnsys.2014.00121

Matos, H. d. C., Koike, B. D. V., Pereira, W. d. S., de Andrade, T. G., Castro, O. W., Duzzioni, M., et al. (2018). Rhythms of Core Clock Genes and Spontaneous Locomotor

Activity in Post-Status Epilepticus Model of Mesial Temporal Lobe Epilepsy. *Frontiers in Neurology* 9. doi:10.3389/fneur.2018.00632

Wallace, E., Wright, S., Schoenike, B., Roopra, A., Rho, J. M., and Maganti, R. K. (2018). Altered circadian rhythms and oscillation of clock genes and sirtuin 1 in a model of sudden unexpected death in epilepsy. *Epilepsia* 59, 1527–1539. doi:10.1111/epi.14513
